# The complete mitogenome of ghost jellyfish *Cyanea nozakii* (Cnidaria, Semaeostomeae, Cyaneidae)

**DOI:** 10.1080/23802359.2017.1422408

**Published:** 2018-01-05

**Authors:** Mustafa Zafer Karagozlu, Jang-Seu Ki, Yoseph Seo, Chang-Bae Kim

**Affiliations:** Department of Biotechnology, Sangmyung University, Seoul 03016, Korea

**Keywords:** Cnidaria, Scyphozoa, Cyaneidae, complete mitogenome, *Cyanea nozakii*

## Abstract

The cnidarian jellyfishes are impressive organisms to show animal mitochondrial genomic diversities. Their mitogenome structure is linear and tRNA content has one or two in numbers, which is highly different than other metazoans. In this study, a complete mitogenome of the ghost jellyfish *Cyanea nozakii* (Cnidaria, Semaeostomeae, Cyaneidae) was sequenced and analyzed. The mitgenome is 17,381 bp long with 38.5% A, 16.0% C, 13.9% G, and 31.6% T nucleotide distributions. In addition, phylogenetic relationship of *C. nozakii* in the class Scyphozoa was investigated by using mitochondrial protein coding genes. Due to results, *C. nozakii* was positioned in the paraphyletic order Semaeostomeae. This is the first complete mitogenome from the genus *Cyanea.*

Ghost jellyfish *Cyanea nozakii* is a scyphozoan species which belongs to the family Cyaneidae. They are mainly found in northeastern parts of the East China Sea and the Yellow Sea (Dong et al. 2008). Economically they are important as a collagen source (Zhang et al. 2014); however, in their blooming season they become risk factor for fishing industry because decomposition of jellyfish strongly influences the marine ecosystem (Qu et al. 2015). Despite economic importance they are not well-studied organisms as much as the other *Cyanea* species (Dong et al. 2008). There is only one mitochondrial genome recorded from the genus and it is partial mitochondrial genome of *C. capillata* (Kayal et al. 2012). The aim of this study is sequencing and analyzing complete mitogenome of *C. nozakii*, and then investigating their genomic structures and molecular phylogeny by using mitogenome proteins.

The specimens of *C. nozakii* have been collected from Tando Bay, Korea (34°59′51.60″N, 126°01′53.01″E). Total genomic DNA was extracted from the jellyfish umbrella by using the cetyl trimethylammonium bromide method (Ausubel et al. 1989) and the remained part of the specimen were stored in Sangmyung University (accession number S001). NGS sequencing was subjected to the gDNA (Miseq, Illumina, San Diego, CA), and paired end reads of mitogenome sequences were assembled and annotated by using MITObim (Hahn et al. 2013) and MITOS (Bernt et al. 2013), respectively. Phylogenetic tree reconstructed based on concatenated amino acid sequences of 13 mitochondrial protein coding genes by using the software MEGA 7.0 (Kumar et al. 2016).

The length of complete mitogenome of *C. nozakii* is 17,381 bp and the GenBank accession no. is MG735260. It consists of 13 protein-coding genes which are *cox1, cytb nad4, nad1, nad4l, nad3, nad6, nad5, nad2, cox3, atp6, atp8, cox2*, two tRNAs (tRNA^Met^, tRNA^Trp^ respectively) and two rRNA genes (large subunit and small subunit respectively). Although, length of the complete mitogenome of the *C. nozakii* was longer than *C. capillata* (16,202 bp) structure and orientation of the genes was identical. The main reason of the size difference in mitogenomes was a long non-coding area which located between *cox1* and *cytb* genes. The all genes encoded on the majority strand except *cox1* in the both mitogenomes. In the mitogenome the genes were using three different starting codons which are ATG, ATT and ATA. On the other hand, there were there were three different stop codons observed which were TAA, TAG and incomplete T(AA).

The phylogenetic relationship of *C. nozakii* in Scyphozoa was analysed by using conducted 13 mitochondrial protein coding genes (Figure 1). Due to results *C. capillata* is the closest species to *C. nozakii* and they are positioned in the paraphyletic order Semaeostomeae. The genus *Chrysaora* is the closest genus to the *Cyanea*. These results are similar with the previous molecular phylogenetic study based on 18S and 28S ribosomal DNA of scyphozoan jellyfish families (Bayha et al. 2010). This study provides additional data of the complete mitogenome of *C. nozakii* to better understand the molecular phylogenetic relationship among Cnidaria species.

**Figure 1.**
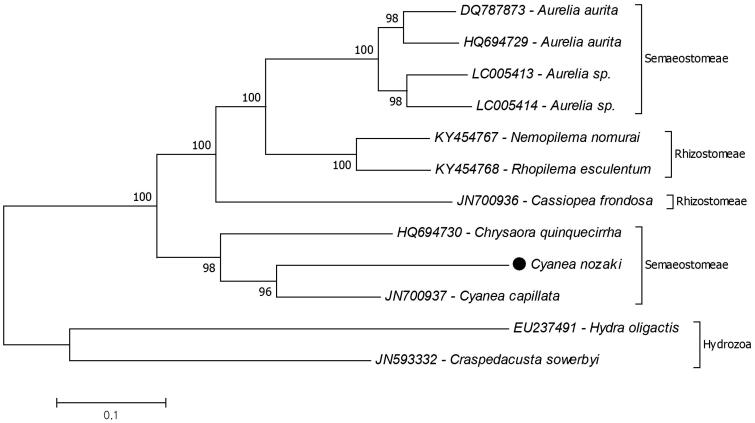
Reconstructed phylogenetic tree of the class Scyphozoa. The scyphozoan mitogenome data retrieved from GenBank and mitogenome of *Cyanea nozakii* (black dot) added. Hydrozoa represents the outgroup.
